# A mathematical model for supercooling process and its application to frazil ice evolution

**DOI:** 10.1038/s41598-023-33097-z

**Published:** 2023-04-10

**Authors:** Deming Yang, Jijian Lian, Xin Zhao, Qingzhi Hou, Yunfei Chen, Yue Zhang

**Affiliations:** 1 State Key Laboratory of Hydraulic Engineering Simulation and Safety, Tianjin University, Tianjin, China; 2 School of Civil Engineering, Tianjin University, Tianjin, China

**Keywords:** Hydrology, Engineering

## Abstract

The calculation of the number of ice crystals for the model of frazil ice evolution is very important and affects the whole frazil events. In this paper, the general formula for the number of frazil ice crystals was established considering secondary nucleation, flocculation, gravity and turbulent entrainment, and ice crystals by melting. Meanwhile, two physical processes of secondary nucleation and flocculation were expressed by introducing critical impact velocity and the probability of flocculation from previous models. It has been found that the simulation results of frazil ice evolution are in good agreement with the experimental data and actual project. Then, Sobol method is carried out to judge parameters’ influence degree, which found the number of nuclei produced $E$ is the most sensitive and has the greatest influence on the model results. In addition, sensitivity analysis of these parameters shows that they can affect the maximum supercooling and the period of supercooling. Therefore, the calculation method of the number of ice crystals is applied, which provides technical support for exploring the water temperature and internal relationship of frazil ice evolution.

## Introduction

For cold regions of the north, frazil ice formation and evolution are seriously affected the operational safety of water conveyance projects and rivers in winter. Affected by the weather, different degrees of icing problems often occurs, which will change the hydraulic conditions and cause damage to water conveyance structures^1–3^, such as blocking pump gates and stations or water intakes of hydroelectric power plants. Frazil ice is the origin of other forms of ice, and it can develop surface ice to form ice cover and accumulate into hanging dams, or attach to river beds and canal bottoms to promote the growth of anchor ice. Frazil ice evolution is a complex physical process, involving the interaction of hydrodynamics, mechanical forces, etc. Therefore, it’s of great significance to research the frazil ice to mitigate or eliminate the aforementioned problems.

Frazil ice evolution is mainly divided into two parts: the number of ice crystals and the heat balance. The former includes multiple physical process, such as initial seeding, secondary nucleation, flocculation, breakup, gravity and turbulent entrainment^4–7^. It is generally believed that frazil ice evolution requires three conditions: turbulence, degree of supercooling and initial seed ice crystals^5^. The first two are necessary conditions for effectively maintaining the process of heat transfer between water and the external environment, while the latter is a key factor in heterogeneous nucleation. Many studies had found that frazil ice will appear and grow around 0 °C in nature^8,9^. In theory, temperature in nucleation never gets close to the critical temperature of heterogeneous nucleation of any known material^10^. Therefore, it is assumed that the initiation of ice crystals originated from the settlement of ice crystals in the air. The settlement of ice crystals is related to snowfall, blowing snow, water drop or bubbles produced by water collision^11,12^. Therefore, secondary heterogeneous nucleation is regard as the main mechanism of nucleation. Once the seed ice crystals appear, it will grow and multiply, and the latent heat released by the growing ice crystals and the heat exchange with the external environment between maintain a dynamic balance. The multiplication of ice crystals is mainly caused by many physical processes, such as the shedding of small ice crystals when ice crystals collide each other or solid boundaries, flocculation between crystals or turbulent shear fragmentation.

The secondary nucleation and flocculation of ice crystals are produced by the collision of solid particles. In fact, the collision of ice particles is very important in many physical systems. Ice particles in the atmosphere transfer electrical charge through collisions^13,14^, which can cause thunderstorms, or the accumulation of dry snow on the surface of cars and roads^15^. For the behavior of ice particles, the commonly used theoretical methods include the Hamaker theory, JKR (Johnson–Kendall–Roberts) model, DMT (Derjaguin Muller Toporov) collision model, etc. These models adopt different assumptions to explain the adhesive interaction of spherical particles due to Van der Waals forces. The JKR contact theory is based on Hertz’s theory, which believes that the adhesion exists in the contact area^16^. And it is applicable to the case where the relative contact radius is large and the adhesion in outside the contact area is relatively small^17–19^. Using JKR theory of adhesive elastic contact to study the interaction between adhesion and inelastic deformation in the impact of elastic solids^20^.

Through a series of research experiments, it is found that the states of ice particles are divided into the unchanged state and the changed state, and present four different degrees of fragmentation after the secondary nucleation^21^. The flocculation of ice particles is divided into low-speed adhesion and high-speed fusion^22^, and the velocity of rivers or channels is generally low, so the ice crystals’ flocculation belongs to the former. In addition, adhesion of ice crystals at low collision velocities has been experimentally demonstrated, and there appears to be a critical velocity beyond which ice crystals no longer stick together^23,24^. The calculation formula of the critical velocity can be derived from the collision model. Blum et al.^25^ and Doan et al.^26^ compared the experimental data with the theoretical value of the model. They found that the relative collision velocity of SiO_2_ grains below 1.2 m/s often leads to adhesion, and rough grains are more prone to adhesion, which is consistent with the prediction of the theoretical model.

Combined with the above research results, a number of mathematical models have been developed to simulate all kinds of physical processes and frazil ice evolution. Daly^27^ established the model of frazil ice formation and evolution based on the continuity equation of ice number and heat balance equation. Then he wrote the report about comprehensive overview of frazil ice^28^, and the dynamic evolution of the crystal size distribution function. Mercier^29^ using Monte Carlo methods and probability density function to simulate frazil ice formation. Base on Daly’s model, Wang^7^ divided the ice particles radius into different intervals to establish heat balance equation and number continuity equation. In these models, the process of secondary nucleation and flocculation is simply described, and its collision theory is not elaborated in detail. Shen et al.^12^ presented a numerical model based on a two-layer ice transport formulation for surface and suspended ice, and verified the calculation result of the model by the field data. Lindenschmidt^30^ and Blackburn et al.^31^ improved the model RIVER to simulate the dynamic processes of frazil ice in river systems with complex flow patterns. Makkonen^10^ established the model for the entire active frazil event. Although the relationship between the sizes of ice particle and heat balance was established, the change process of the number of particles was not well simulated.

The number of particles is difficult to be quantified, because the behavior of collisions between ice crystals is not fully understood. Daly^27^ proposed a theoretical formula for the rate of secondary nucleation based on the kinetics of secondary nucleation. Evans et al.^32^ and Mercier^29^ proposed the model for the kinetics of secondary nucleation, and considered that the collision breeding was the primary mechanism. Then Jucha et al.^33^ found that the impact of turbulence velocity fluctuation becomes the main factor determining the collision rate. Hammar and Shen^5^ simulated secondary nucleation and flocculation based on binary collisions of frazil particles. It can be found from that the quantization of each process is relatively complex, and many uncertain parameters can hardly guarantee the accuracy of model results. In addition, there is a lack of sensitivity analysis of these parameters to determine their position and role in the model.

In this paper, based on previous models and the theory of collision between ice particles, two processes of secondary nucleation and flocculation were expressed by introducing critical impact velocity and probability of flocculation, and general formula for calculating the number of frazil ice crystals was established. Meanwhile, the method were verified though the experimental data and actual project combination with the two-layer ice transport model. Finally, this paper uses Sobol method^34^ to judge parameters’ influence degree and analyzes the sensitivity of parameters to expound the influence of the supercooling process, which provides technical supports for the verification of model parameters.

## Modelling

According to the available research results, in order to express the parameters clearly and calculate the model conveniently, in this paper, the frazil ice crystal is regarded as a uniform size of disc-shaped. The mean diameter of frazil ice crystals is used as the calculated size of all ice crystals. Also, assumed that the space of model is fully homogeneous and there is no difference with location.

### The number of ice crystals

Based on Daly’s^27^ general equation and Svensson and Omstedt’s^35^ model, particles breakup is generally not considered because it only exists in large, weakly bounded aggregates at high shear^29^.

Before the water is supercooled no crystals can exist in the water, in fact, new crystals are introduced by seeding, ice crystals that fall into the water from the atmosphere. Therefore, the change of the number of ice crystals in the period of supercooling only considers five physical processes, namely initial seeding, secondary nucleation, flocculation, gravity and turbulent entrainment, and ice crystals by melting. The general formula that can calculate the number of frazil ice crystals are considered as

1 $$\frac{dn}{dt} = {{n}_{\mathrm{seed}}+n}_{\mathrm{colli}}-{n}_{\mathrm{floc}}-{n}_{\mathrm{g}+\mathrm{t}}-{n}_{\mathrm{melt}},$$

where $n$ = the number of frazil ice crystals per unit volume; ${n}_{\mathrm{seed}}$ = the initial seeding rate; ${n}_{\mathrm{colli}}$, ${n}_{\mathrm{floc}}, {n}_{\mathrm{g}+\mathrm{t}}, {n}_{\mathrm{melt}}$ = the number of crystals produced by secondary nucleation, flocculation, gravity and turbulent entrainment, and ice crystals by melting per time per unit volume. And ${n}_{\mathrm{melt}}$ is closely related to the thermal balance of frazil growth, and the specific process is described in “The thermal balance of frazil growth” section.

#### Secondary nucleation

Secondary nucleation and flocculation are both caused by the collision of ice crystals. Secondary nucleation is responsible for producing small particles by removing the nuclei created from the surface of the parent particles. Evans et al.^32^ and Mercier^29^ proposed the model for the kinetics of secondary nucleation, and it assumed the collision frequency was related to turbulent shear and differential rising. The number of particles produced by the collision of two identical ice crystals is

2 $$I = \iint E{C}_{E}d{v}^{2},$$

where $v$ = one of the colliding ice crystals; $I$ = the number of nuclei produced per unit time; $E$ = the number of nuclei produced per unit collision energy; ${C}_{E}$ = the rate of collisional energy transfer to the crystals per unit volume of fluid, ${C}_{E}$ is a function of collision frequency and collision efficiency.

**Figure 1:**
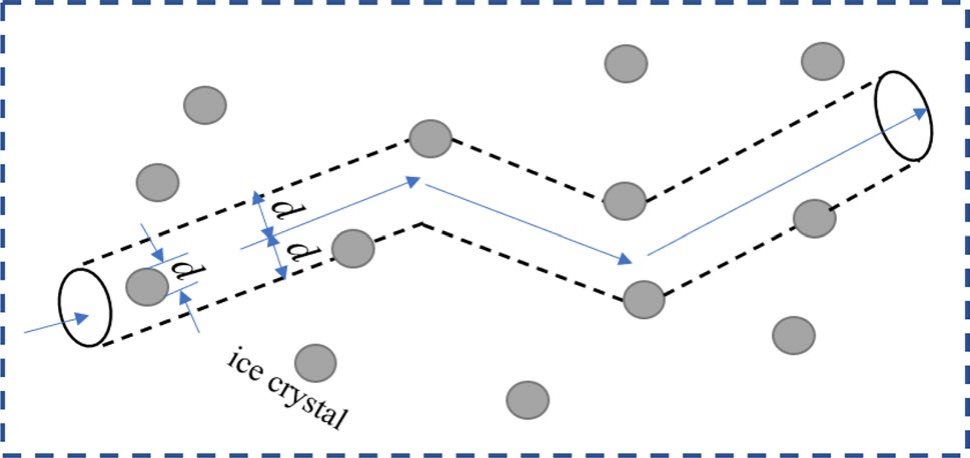
Schematic diagram of the collision zone between ice crystals.

Due to the ${C}_{E}$ are not easy to solve, it is simplified on this basis and combined with the random theory, take the trajectory of any ice crystal as the central axis, and the effective diameter of the crystal as the radius to make a cylinder (as shown in Fig. 1). In unit time $\Delta t$, the relative velocity of ice crystals is $\overline{u }$. The distance traveled by ice particles is $\overline{u }\Delta t$, the volume of the corresponding cylinder is $\uppi {d}^{2}\overline{u }$. Since the ice crystal density per unit volume is $n$, then the average collision frequency of each ice crystal is

3 $$\overline{Z } =\uppi n{d}^{2}\overline{u },$$

where $d$ = the diameter of ice crystals; $\overline{u }$ = the relative velocity, $\overline{u } = \sqrt{{u}_{l}^{2}+{w}^{2}}$, where ${ {u}_{l} = \left(1/15\right)}^{1/2}{\left(\varepsilon /\vartheta \right)}^{1/2}d$, is used as the velocity due to turbulent fluctuation^7^; $w$ = the average velocity of rising frazil ice; $\varepsilon$ = turbulent dissipation rate; $\vartheta$ = coefficient of kinematic viscosity.

According to the model established by Mercier^29^, assuming that the proportion of collisional energy transfer to the crystals per unit volume is $\tau$. ${n}_{\mathrm{colli}}$ can be formulated as

4 $${n}_{\mathrm{colli}} = \int\uppi {n}^{2}{d}^{2}\overline{u }\times E\times \tau \times m{\overline{u} }^{2}dt = \int \frac{{\uppi }^{2}}{4}{\rho }_{i}SE\tau {n}^{2}{d}^{5}{\overline{u} }^{3}dt,$$

where $m$ = the mass of ice crystal, $m = \frac{\pi {\rho }_{i}S{d}^{3}}{4}$; the aspect ratio $S = \delta /d$; $\delta$ = the thickness of ice crystal; ${\rho }_{i}$ = the density of ice. According to the research results of Hill et al.^24^, it is found that after millimetre-sized ice particles collide with each other, coefficients of restitution were evenly spread from 0.08 to 0.65, and 0.08% to 17%, 4% to 41% of the kinetic energy was converted into the particles’ rotation and translation. Therefore, the value range of the parameter $\tau$ is 0–0.88, so the average value $\tau$ = 0.44 is taken in this paper.

Growing frazil ice crystals can release latent heat to make the water temperature increase and the growth rate of ice decrease. An active frazil event involves a negative feedback mechanism. This puts a limit on the amount of ice that can form, given the time and the heat flux from the water body. So it is quite logical to limit $n$ by the calibration factor ${n}_{\mathrm{max}}$ to restrict, then

5 $$n = \text{min}\left({n}_{0},{n}_{\mathrm{max}}\right).$$

Before the water is supercooled no crystals can exist in the water, therefore ${n}_{0}$ must always be zero. When the water is supercooling, ${n}_{0}$ is related to the initial seeding rate. $E$, ${n}_{\mathrm{seed}}$, ${n}_{\mathrm{max}}$ are calibration parameter, at the same time, the selection of these can refer to Ye et al.^35^, Clarks^36^, Wang^7^, etc.

#### Flocculation

Flocculation is an adhesion phenomenon caused by the adhesion of contact surfaces^19,23^. The flocculation between ice crystals is closely related to the thermal conditions determined by water temperature. It is necessary for the temperature of the water to be less than 0 °C for flocculation to occur.

**Figure 2:**
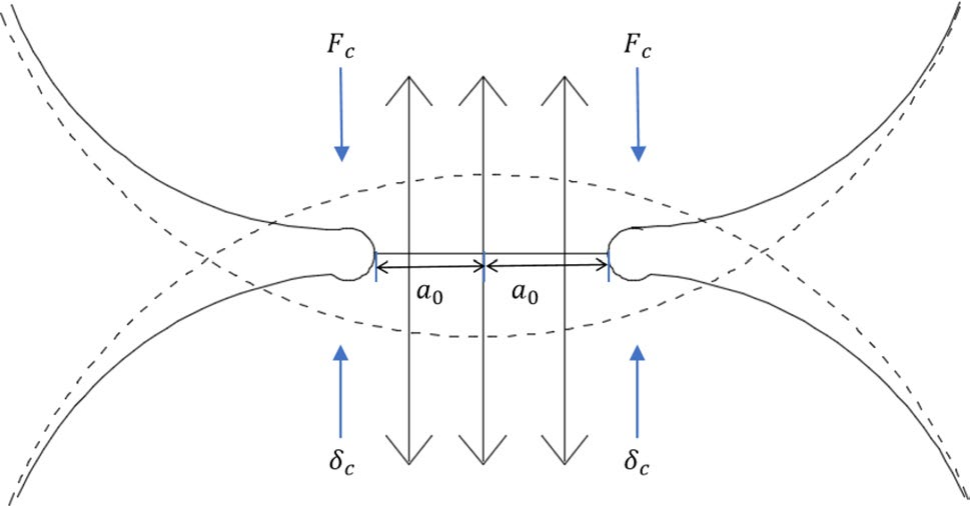
Contact diagram between particles in JKR contact theory.

Svensson and Omstedt^37^ suggested the probability of flocculation is linearly related to the diameter of colliding particles. Some studies show that the adhesion between particles is related to the collision velocity, but most of the critical adhesion velocity is calculated in the air fluid. Although the ice crystal collision exists in the liquid fluid, there may be some differences. However, JKR theory, whose research object is collision contact surface, can effectively avoid the influence of fluid force. According to the JKR theory (shown in Fig. 2) and the generalized regime map by conducting experiments^38^, the critical pull-off force ${F}_{c}$ of adhesion on the contact surface of two particles is given by

6 $${F}_{c} = \frac{3\pi W{R}^{*}}{2},$$

where $W$ = work of adhesion, $W$ = 0.218 J/m^2^; ${R}^{*}$ = the effective radius of contact, ${R}^{*} = \frac{d}{4}$.

Then, the corresponding contact radius ${a}_{0}$ and particle critical overlap ${\delta }_{c }$ are

7 $${a}_{0} = {\left(\frac{9\pi W{R}^{*}}{2{E}^{*}}\right)}^{1/3},$$

8 $${\delta }_{c} = \frac{{a}_{0}^{2}{R}^{*}}{2}-\sqrt{\frac{2\pi W{a}_{0}}{{E}^{*}}},$$

where ${E}^{*}$ = effective Young’s modulus.

The energy loss caused by the collision of two ice crystals is equal to the work done by the unloading force, which can be obtained by numerical integration along the path of over lap

9 $${E}_{s} = \underset{0}{\overset{{a}_{0}}{\int }}{F}_{c}d\delta = {K}_{1}{F}_{c}{a}_{0} = \frac{9{K}_{1}}{8}{\left(\frac{{\pi }^{5}{W}^{5}{{R}^{*}}^{4}}{{{E}^{*}}^{2}}\right)}^{1/3},$$

where ${E}_{s}$ = energy to stick at collision; ${K}_{1}$ ≈ 0.9355 is an integration constant.

According to the conservation of energy, the critical adhesion velocity ${V}_{s}$ is

10 $${V}_{s} = {\left(\frac{2{E}_{s}}{{m}^{*}}\right)}^{0.5} = {\left(\frac{9{K}_{1}}{4{m}^{*}}\right)}^{0.5}{\left(\frac{{\pi }^{5}{{{W}^{5}R}^{*}}^{4}}{{E}^{{*}^{2}}}\right)}^{1/6},$$

where ${m}^{*}$ = effective mass in contact.

In order to better reflect the process of flocculation between ice crystals, combined with the critical adhesion velocity^18,39^, the probability of flocculation $\beta$ is introduced. At low collision velocities, the ice crystals have high adhesion to each other and may be “trapped” without bouncing back^17,20,40^. Thus $\beta$ can be defined as,

11 $$\beta = \left\{\begin{array}{l}1, \quad \overline{u}\le {V }_{s} \\ 0, \quad \mathrm{ others }\end{array}.\right.$$

Base on the average collision frequency $\overline{Z }$, the number of flocculation produce ice crystals is defined as,

12 $${n}_{\mathrm{floc}} = \int\uppi {\beta n}^{2}{d}^{2}\overline{u } \,\,dt.$$

#### Gravity and turbulent entrainment

Mercier^29^ and Wang^7^ had calculated the removal coefficients by assuming that the removal efficiency was positively correlated with the concentration of frazil ice. Chen and Shen^41^ used the probability to express the process of gravity and turbulent entrainment. It can be seen from previous research results that ice crystals that move upward due to buoyancy are removed when they become part of the top layer, and they that move downward are removed when they become part of the anchor ice at the bottom of the channel. Assuming that the depth of the mixed region is homogeneous, the calculation formula for removal is

13 $${n}_{\mathrm{g}+\mathrm{t}} = {P}_{\mathrm{g}+\mathrm{t}}n = \frac{(w+{w}_{d})}{H}{M}_{i}n,$$

where ${P}_{\mathrm{g}+\mathrm{t}}$ = the removal coefficient of gravity and turbulent entrainment, ${P}_{\mathrm{g}+\mathrm{t}} = \frac{(w+{w}_{d})}{H}{M}_{i}$; ${M}_{i}$ = the concentration of frazil ice per unit volume; ${w}_{d}$ = the average downward velocity due to turbulent entrainment; $H$ = the depth of river or channel.

Based on the above assumptions, the volume concentration of frazil ice per unit volume ${M}_{i}$ is

14 $${M}_{i} = \frac{1}{4}n\pi S{d}^{3}.$$

Equation (13) gives

15 $${n}_{\mathrm{g}+\mathrm{t}} = \int \frac{(w+{w}_{d})}{4H}{n}^{2}\pi S{d}^{3}dt,$$

### Frazil ice evolution

#### The aspect ratio $S$

The aspect ratio is closely related to the growth of ice crystal, due to the crystal wants to minimize its surface-to-volume ratio, so the cross growth of diameter is superior to vertical growth^27,42^. With the decrease of water temperature, the aspect ratio also gradually decreased until it became stable. Some researchers found that the aspect ratio is related to the rise velocity $w$ of frazil ice, and it changes within a small ^28,38,43^, because the heat transfer from the ice crystals is also a function of the rise velocity. Gosink and Osterkamp^44^ derived the equation of rising velocity of a disc-shaped ice crystals as follows:

16 $$S = \frac{{w}^{2}{C}_{D}}{2d{g}{{^{\prime}}}},$$

where ${C}_{D}$ = the drag coefficient; ${g}{^{\prime}}$ is the reduced gravitational acceleration, given by ${g}{^{\prime}} = g\left({\rho }_{w}-{\rho }_{i}\right)/{\rho }_{w}$.

In addition, there are many methods for calculating the rise velocity, but some applicable conditions may exist. Therefore, the three equations derived^17,45^ is adopted as follows:

17 $$w = 0.02 \left({g}_{D}^{\prime}{\vartheta }^{-1}{d}^{2}\right) \, {\text{for}} \, d < 0.0006 \, {\text{m}},$$

18 $$w = 0.0726 \left({{g}_{D}^{\prime}}^{0.715}{\vartheta }^{-0.428}{d}^{1.14}\right) \, {\text{for}} \, 0.0006<d<0.0028 \, {\text{m}},$$

19 $$w = \frac{1}{2}{\left({g}_{D}^{\prime}d\right)}^{1/2} \, {\text{for}} \, d\ge 0.0028 \, {\text{m}},$$

where ${g}_{D}^{{\prime}}$ = the reduced gravity.

According to previous studies, it is assumed that the aspect ratio is in the range of $\left[\mathrm{0.01,0.1}\right]$.

**Figure 3:**
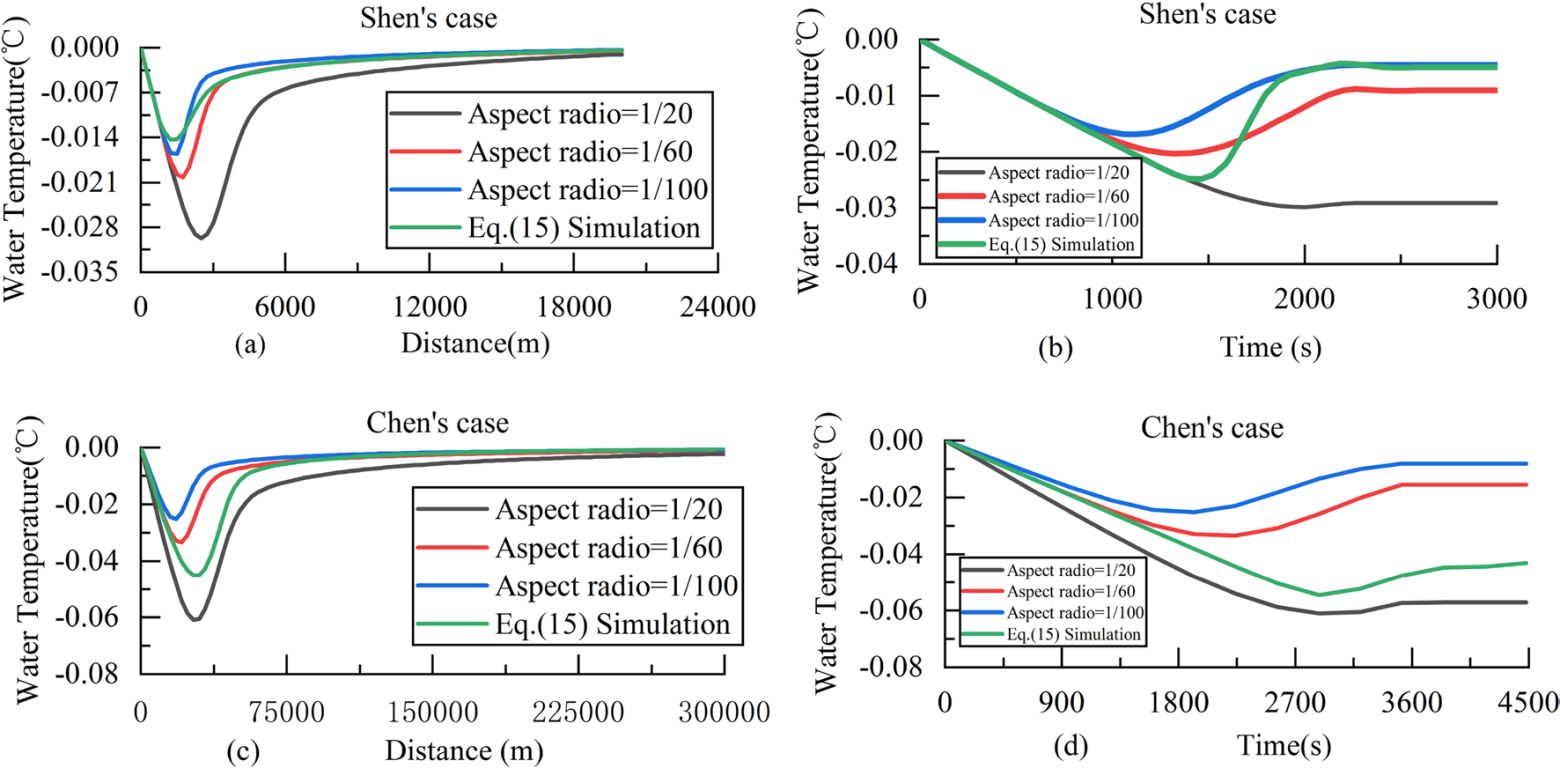
(**a**) Water temperature along the lines for Shen’s case. (**b**) Water temperature versus time for Shen’s case. (**c**) Water temperature along the lines for Chen’s case. (**d**) Water temperature versus time for Chen’s case.

Equations (16)–(19) was introduced into the two-layer ice transport model to verify the cases in Shen^46^ and Chen^41^, so as to understand the influence of the aspect ratio on the model results. Compares the results that the aspect ratio is set as a constant value, which were respectively 1/20, 1/60 and 1/100. The initial condition and simulation results are shown in Table 1 and Fig. 3.

**Table 1:** Initial condition of Shen’s^46^ case and Chen’s^41^ case.

Parameter name	Shen’s case	Chen’s case
Width	100 m	425 m
Flow area	500 m^2^	1350 m^2^
Length	20 km	300 km
Rising velocity	0.001 m/s	0.0013 m/s
Surface heat loss rate	360 W/m^2^	431W/m^2^
The initial diameter	0.0005 m	0.001 m
The initial seeding rate	7.5E3	4.8E5
Porosity of the ice flower	0.5	0.6
Thickness of the hard ice layer	0.005 m	0.3 m
The number of nuclei produced per unit collision energy	3E12	3E14

Figure 3 show that the aspect ratio changes dynamically during the supercooling process, and directly affects the change of water temperature. It also can be seen from Fig. 3a,c that the water temperature along the lines for Shen’ case is between the results of aspect ratio of 1/20–1/60, while the water temperature for Chen’ case is between 1/60 and 1/100, so it is concluded that the influence of aspect ratio on water temperature along the lines is different under different conditions. With the decrease of the aspect ratio $S$, the maximum supercooling decreases in Fig. 3b,d. Therefore, using the Eq. (16) to calculate the aspect ratio can better reflect the actual process and reduce the errors.

#### The thermal balance of frazil growth

According to the thermal balance process of frazil ice growth, the equation is

20 $$\frac{d{T}_{w}}{dt} = \frac{1}{\mathrm{c}}\left({L}_{i}\frac{d{M}_{i}}{dt}-\frac{\varphi }{H}\right),$$

where ${T}_{w}$ = water temperature; $c$ = the volumetric specific heat of water;${L}_{i}$ = the volumetric latent heat of ice;$\varphi$ = the heat flux per unit area from the water surface to air.

Differentiating Eq. (20) gives

21 $$\frac{d{M}_{i}}{dt} = \frac{3}{4}n\pi \delta d\frac{\mathrm{d}\left(d\right)}{\mathrm{dt}}.$$

The latent heat flux released by the growth of all particles is equal to the heat flux into water, so that.

22 $${\rho }_{i}{L}_{i}\frac{dV}{dt} = h\Delta T{A}_{i},$$

where $V$ = the volume of the disk; $h$ = the total heat transfer coefficient of the ice crystal, $h = \frac{kNu}{d}$; $k$ = the conductivity of heat in water and $Nu$ = the Nusselt number; $\Delta T$ = the difference of between water temperature and freezing point; ${A}_{i}$ = the area of ice crystals.

The Nusselt number is a dimensionless number related to Prandtl number ($Pr$ = 13.4) and Reynolds number that characterizing heat diffusion.

23 $$Nu = C{Pr}^{1/3} \,{Re}^{m}.$$

The values of constant $C$ and $m$ are also different in different flow states. According to the actual engineering and Kobus and Shumway’s^47^ classification standard, choose $C$ = 2.22, $m$ = 0.34. Simultaneous Eqs. (20)–(23) to obtain an equation for the growth rate of frazil particle diameter as.

24 $$\frac{\mathrm{d}\left(d\right)}{\mathrm{dt}} = \frac{4}{3}\left(\frac{d}{2\delta }+1\right)\frac{h\Delta T}{{\rho }_{i}{L}_{i}}.$$

## Results

### Model verification

**Figure 4:**
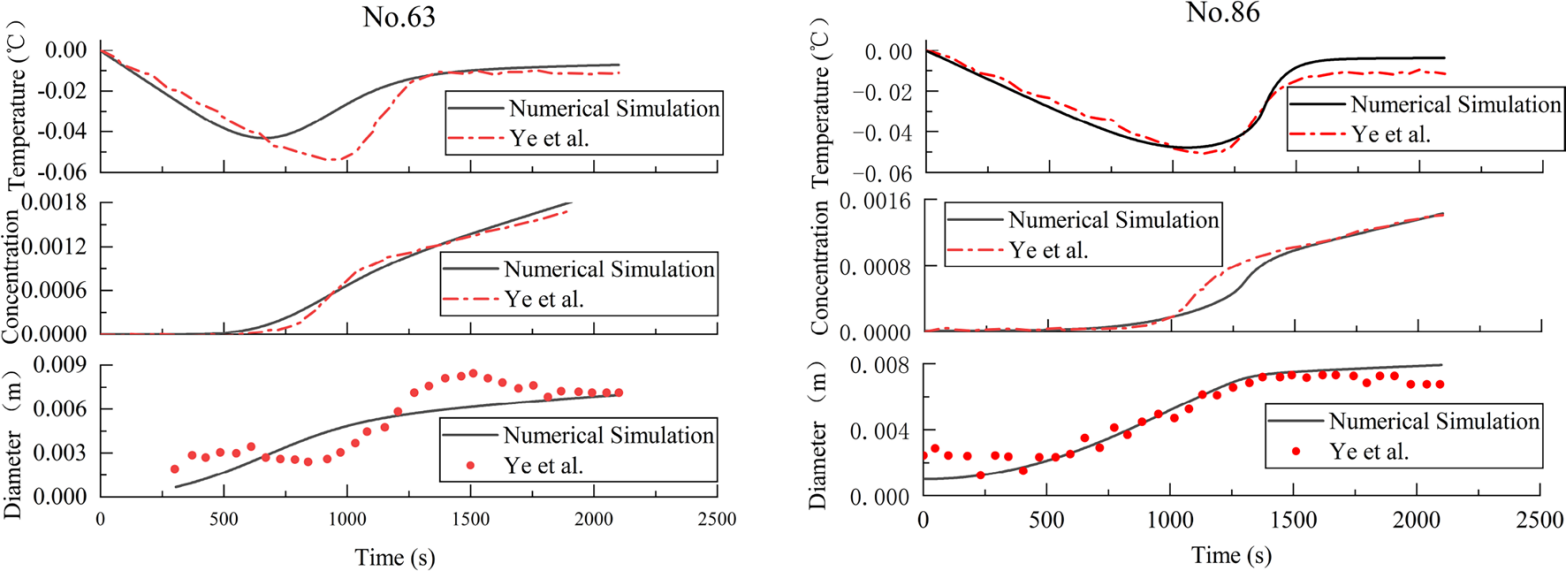
Water temperature, frazil ice concentration and ice crystal diameter measured in the laboratory experiment no. 63 and no. 86 by Ye et al. and as simulated by the model.

Various experimental studies on frazil ice formation have been carried out in different laboratory setting. By changing the hydraulic and environmental conditions that include water depth, and water velocity, air temperature, different working conditions are simulated, so as to explore the variation law of each factor in the whole supercooling process. In this paper, experimental data of Ye et al.^35^ were used to verify the accuracy and reliability of the model. Selecting the laboratory experiment no. 63 and no. 86 by Ye et al. as the examples and their characteristics of the supercooling processes shown in the Table 2. The two groups of experiments use different water velocity and water depths, which can better simulate the process of frazil ice evolution under different conditions. Finally, using the model to calculate the supercooling process of the two examples, the results are shown in the Fig. 4.

**Table 2:** Characteristics of the supercooling processes for the laboratory experiment no. 63 and no. 86 by Ye et al.

Exp. no.	Air temperature (°C)	Velocity (m/s)	Water depth (m)	The heat flux (W/m^2^)	${n}_{seed}$	${n}_{max}$
63	− 10	0.612	0.15	40	7.5E2	1.5E4
86	− 10	0.483	0.2	40	1.5E3	3.5E4

Then, using Eqs. (25) and (26) to calculate *MAE* (mean absolute error) and *RMSE* (root mean square error), the calculation results of each parameter are shown in Table 3.

25 $$MAE = \frac{1}{s}\sum_{i = 1}^{s}\left|{y}_{s}^{i}-{y}_{r}^{i}\right| ,$$

26 $$RMSE = \sqrt{\frac{1}{s}\sum_{i = 1}^{s}{\left({y}_{s}^{i}-{y}_{r}^{i}\right)}^{2}},$$

where ${y}_{s}^{i}$, ${y}_{r}^{i}$ = the measure and simulation value of parameters $i$;$s$ = the quantity of data.

**Table 3:** *MAE* and *RMSE* of water temperature, frazil ice concentration and ice crystal diameter in the laboratory experiment no. 63 and no. 86.

Exp. no.	Index	Water temperature	Concentration	Diameter
63	*MAE*	0.0041	4.6E−5	0.00113
	*RMSE*	0.0047	6.5E−5	0.00130
86	*MAE*	0.0030	1.39E−5	0.00055
	*RMSE*	0.0033	1.69E−5	0.00072

Figure 4 shows with the increase of ice crystal diameter and frazil ice concentration, the water temperature versus time will reach the peak and then change until it is stable, which is consistent with the theoretical supercooling process. It can found water temperature reaches its lowest point at about 600 s and 1200 s. Thereafter, increased latent heat liberation leads to an increase in the water temperature until the residual temperature at 1300 s and 1600 s, which marks the beginning of the residual supercooling stage. In addition, Person correlation coefficient between the concentration of frazil ice and the diameter of ice crystals are 0.95 and 0.89, which can be inferred a positive correlation each other.

Table 3 show the *MAE* and *RMSE* of the three parameters in the two experiments are very small, indicating that the errors with the measure values are small and have good agreement with them. The model can better explain evolution of frazil ice concentration and water temperature. Through comparison, it is found that the *MAE* and *RMSE* of no. 86 is significantly lower than that of no. 63, it be due to inaccuracy in the empirical equations of the model or artificial errors in experiments. The Person correlation coefficient between water temperature and concentration are 0.62 and 0.16, diameter and concentration are 0.86 and 0.88. It suggests that water temperature and concentration is poorly correlated, and concentration and diameter is well correlated.

### Applied to actual project

**Figure 5:**
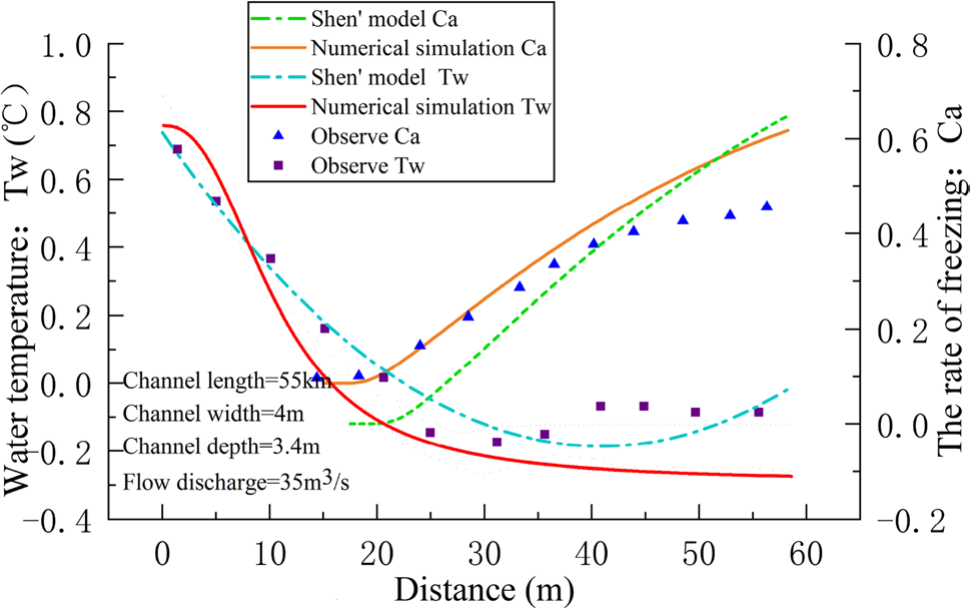
Water temperature and the rate of freezing at January 8(th), 3:00, 2012.

According to the characteristics of various models and the actual situation of the projects, the two-layer ice transport model proposed by Shen et al.^41^ can not only simulate supercooling, suspended ice, surface ice discharges and other processes, but also consider the influence of ice crystal quantity in the conservation of thermal energy. In order to further verify the applicability of the general formula, based on the two-layer ice transport formulation model, this paper simulates the ice-water two-phase flow of channel. Water conveyance projects have been built in the cold areas in northwest China. In winter, the open-flow ice-water two-phase water transfer mode is adopted mostly to alleviate the problem of water shortage. Taking an open ice-water two-phase channel in Xinjiang as example, in winter 2003-01, observations on water temperature and the rate of freezing were made. Taking January 8(th) as a typical temperature process, other parameters do not change, the general formula for the number of frazil ice crystals only is introduced into Shen’s model to simulate the ice process at 3:00, and the calculation results are compared with those Shen’ model was used, as shown in Fig. 5.

The water temperature and the rate of freezing along the lines are shown in Fig. 3. Using ${h}_{ai} = 19.71 \, {\text{W}}/{\text{m}}^{2} \, ^\circ{\rm C}$, the floating probability of the frazil ice $\theta = 0.6$, the porosity of the ice floe ${e}_{f} = 0.5$, the frazil rise velocity $w = 0.001$. No anchor ice formation is assumed. The *MAE* and *RMSE* of water temperature and the rate of freezing for the two model are calculated, as shown in Table 4.

**Table 4:** *MAE* and *RMSE* of water temperature and the rate of freezing for the two methods.

Method	Index	Water temperature	The rate of freezing
Improved model	*MAE*	0.0431	0.0497
	*RMSE*	0.0514	0.0565
Shen model	*MAE*	0.0861	0.0747
	*RMSE*	0.0973	0.1000

The numerical simulation results introduced the general formula are in agreement with the field observations in Fig. 5. And Table 4 show the *MAE* and *RMSE* of water temperature and the rate of freezing using this method is smaller, which proves the feasibility of the formula. The *MAE* and *RMSE* of introducing the general formula are smaller than those of Shen’ model, which it further shows that the general formula for the number of frazil ice crystals can improve the accuracy of simulation results to a certain extent.

## Discussion

### Sobol method analysis

In the process of simulation, although the selection of some parameters refer to some relevant literature, it will still have a certain impact on the simulation results. In order to better understand the role of these parameters, special methods should be adopted for analysis. Sobol method is a global sensitivity analysis method based on variance decomposition. Its core is to decompose the total variance of the objective function into the variance of a single parameter and the variance generated by the interaction between parameters by Nandakumar et al.^48^ Suppose the model can be represented as $y = f\left(x\right) = f\left({X}_{1}, \dots ,{X}_{m}\right)$, where $y$ is the objective function of model,${X}_{m}$ is the model parameters, and $\mathrm{m}$ is the total number of parameters. Then the variance $D\left(\mathrm{y}\right)$ can be decomposed into:

26 $$D\left(y\right) = \sum_{i}{D}_{i}+\sum_{i<j}{D}_{ij}+\cdots +{D}_{\mathrm{1,2},\dots m},$$

where ${D}_{\mathrm{i}}$ is the variance of parameters $i$; ${D}_{\mathrm{ij}}$ is the variance generated by the interaction of the parameters $i,j$; ${D}_{\mathrm{1,2},\dots \mathrm{m}}$ is the variance generated by the interaction of the $\mathrm{m}$ parameters. The sensitivity of each parameter and the sensitivity of the interaction between the parameters can be obtained by normalization.

27 $$1 = \sum_{i}\frac{{D}_{i}}{D\left(y\right)}+\sum_{i<j}\frac{{D}_{ij}}{D\left(y\right)}+\cdots +\frac{{D}_{\mathrm{1,2},\dots m}}{D\left(y\right)}.$$

For parameters, the first-order, second-order and total sensitivity are respectively: ${S}_{\mathrm{i}}$ = $\frac{{D}_{\mathrm{i}}}{D}$; ${S}_{\mathrm{ij}}$ = $\frac{{D}_{\mathrm{ij}}}{D}$; ${S}_{\mathrm{Ti}}$ = 1 − $\frac{{D}_{\sim \mathrm{i}}}{D}$, where $\frac{{D}_{\sim \mathrm{i}}}{D}$ is the variance of parameters other than parameter $i$.

**Figure 6:**
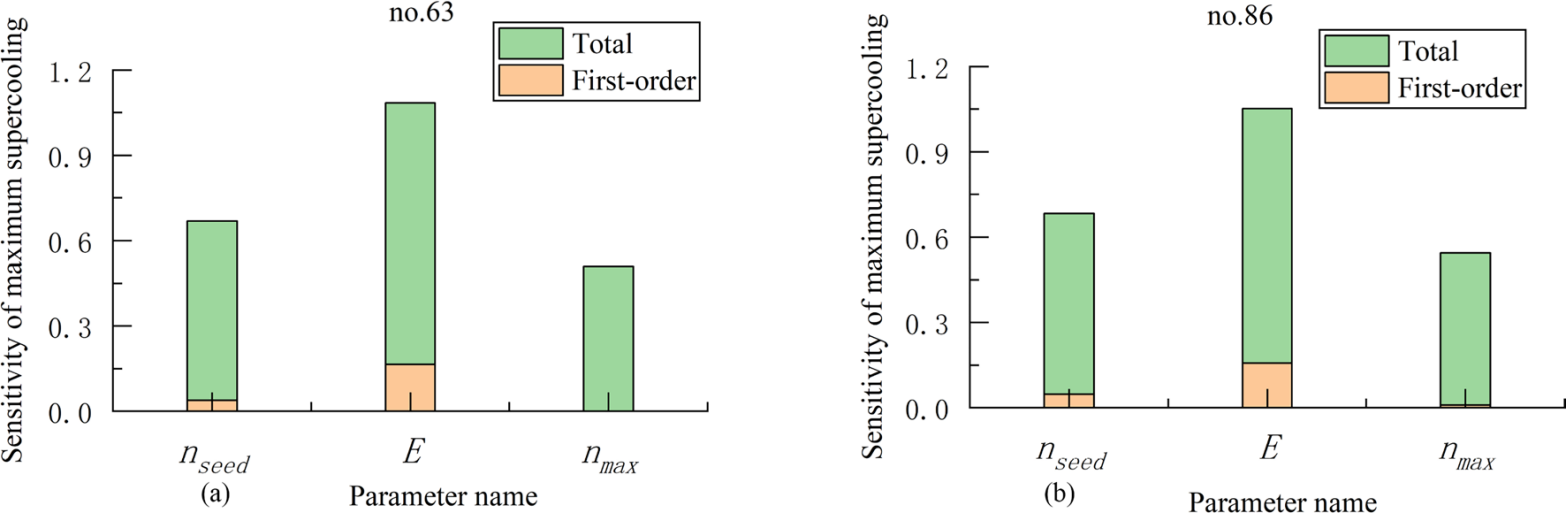
(**a**) The results of the Sobol analysis for three model parameters ${n}_{seed}$, $E,{n}_{max}$ in the experiment no. 63. (**b**) The results of the Sobol analysis for three model parameters ${n}_{seed}$*,* $E,{n}_{max}$ in the experiment no. 86.

According to above model, the maximum supercooling is selected as the objective function, and the three model parameters:${n}_{\mathrm{seed}}$, $E,{n}_{\mathrm{max}}$ are selected for Sobol analysis, the results are shown as Fig. 6.

Figure 6 shows that the green part of the Figure is the total sensitivity of parameters; the yellow part is the first-order sensitivity. That is, the sum of the two parts is the total sensitivity of the parameter. It can be seen that, the sensitivity of $E$ is the largest, reaching 92% and 89% which means that it has the greatest influence on the maximum supercooling. While the sensitivity of ${n}_{\mathrm{max }}$ is the smallest, the first-order sensitivity is basically close to 0, and its total sensitivity is also the smallest, both reaching about 50% which is less than the others. Therefore, when selecting the model parameters, the parameters $E$ should be checked more accurately.

### Sensitivity analysis

Sensitivity analysis is one of the common methods to analyze uncertainty. Hence, the sensitivity analysis of three parameters—${n}_{seed}$, ${n}_{max}$ and $E$ is carried out for the Ye’ experiment to calculate the additional numerical simulations.

**Figure 7:**
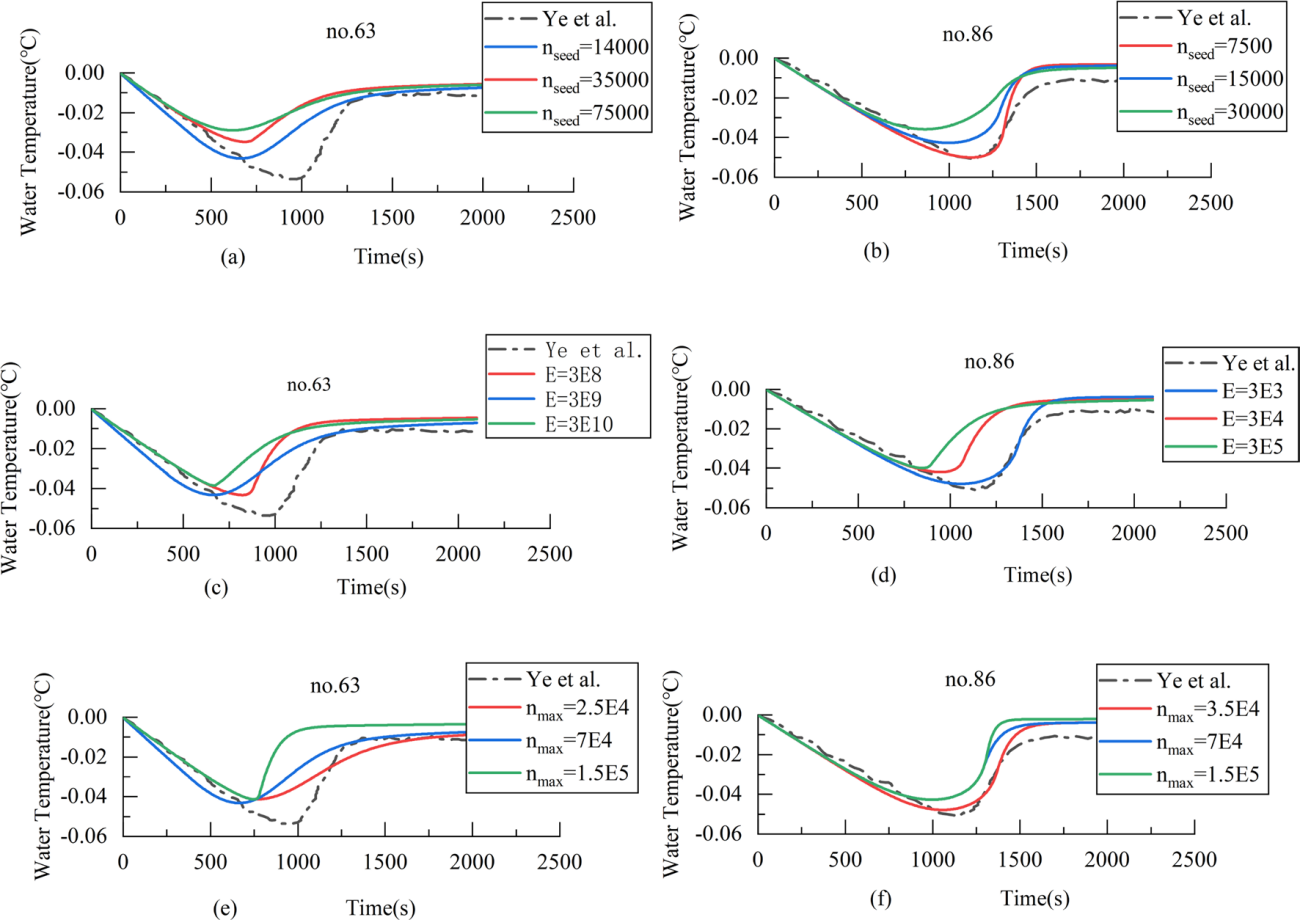
(**a**) Sensitivity analysis of ${n}_{seed}$ for the laboratory experiment no. 63. (**b**) Sensitivity analysis of ${n}_{seed}$ for the laboratory experiment no. 86. (**c**) Sensitivity analysis of $E$ for the laboratory experiment no. 63. (**d**) Sensitivity analysis of $E$ for the laboratory experiment no. 86. (**e**) Sensitivity analysis of ${n}_{max}$ for the laboratory experiment no. 63. (**f**) Sensitivity analysis of ${n}_{max}$ for the laboratory experiment no. 86.

In the above two experiments for no. 63 and no. 86, keeping other parameters unchanged, the different values of ${n}_{seed}$, ${n}_{max}$ and $E$ used in the simulations, as shown in Table 5, and the results are shown in Fig. 7.

**Table 5:** Parameters ${n}_{seed}$, ${n}_{max}$ and $E$ for the laboratory experiment no. 63 and no. 86 by Ye et al.

Exp. no.	${n}_{seed}$			${n}_{max}$			$E$		
No. 63	1.4E4	3.5E4	7.5E4	2.5E4	7E4	1.5E5	3E8	3E9	3E10
No. 86	7.5E3	1.5E4	3E4	3.5E4	7E5	1.5E5	3E3	3E4	3E5

Figure 7a,b shows with the initial number of ice crystal ${n}_{seed}$ increase, the maximum supercooling will be decreased, and the period of supercooling also be shortened. This is basically in line with Wang’s view. For Fig. 7c,d, it also can be seen that its conclusion is similar to Fig. 7a,b, but the magnitude of its influence is significantly less than that of parameter ${n}_{seed}$. This happens to coincide with Fig. 6, which the effect of parameter ${n}_{0}$ on model results is greater than that of parameter ${n}_{max}$. The number of nuclei produced $E$ affect the frequency of collisions between ice crystals in Fig. 7e,f. The higher its value, the more ice crystals the collision produced, the shorter the period of supercooling and the faster the water temperature recovered. The parameters $E$ are greatly affected by the Reynolds number and the dissipation rate^21,29^. Due to the large difference in Reynolds numbers between the two experiments for no. 63 and no. 86, so the magnitude of parameter values varies greatly.

### Discussion on the effect of parameters

**Figure 8:**
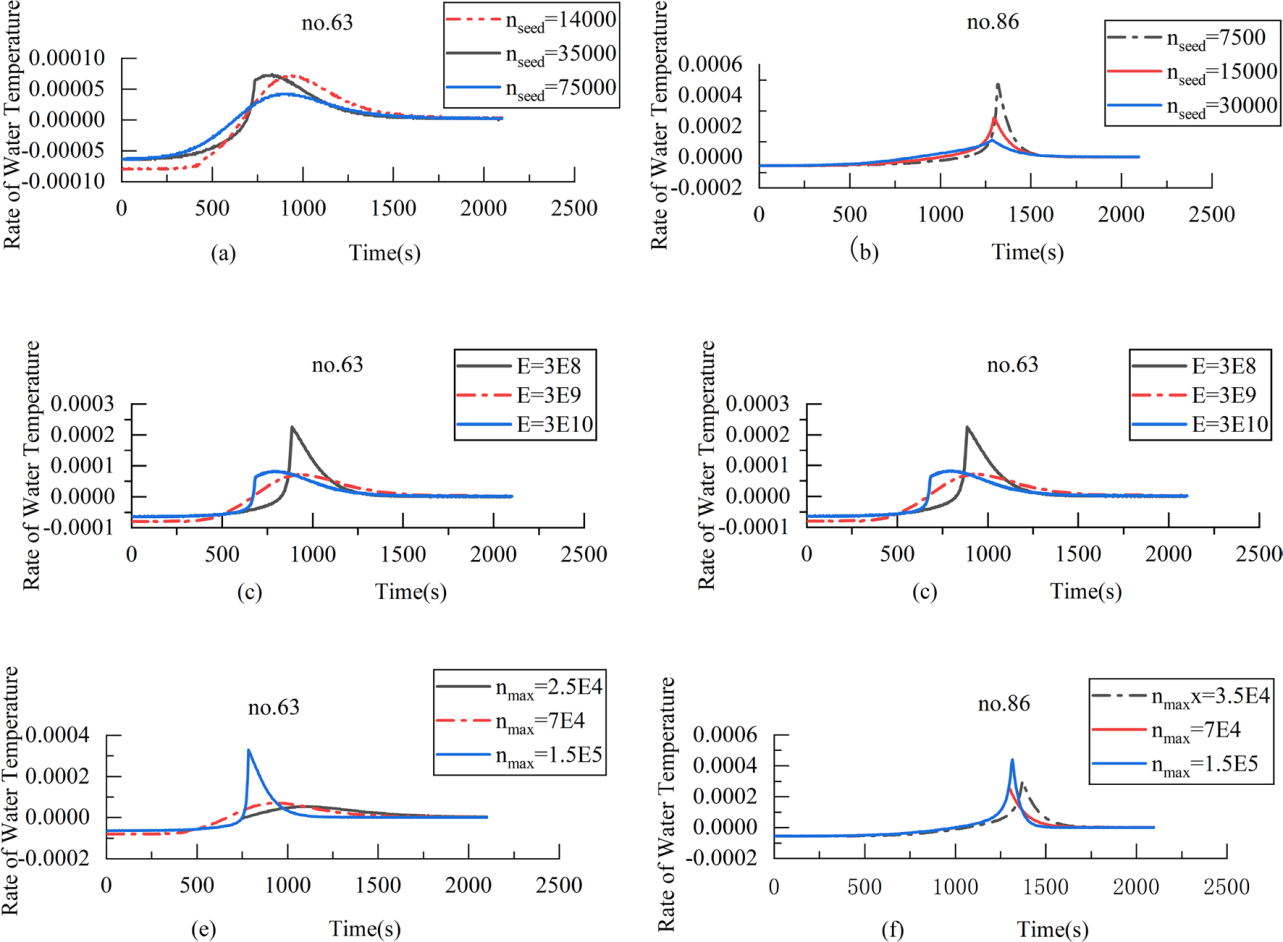
(**a**) Rate of water temperature versus time of ${n}_{seed}$ for the laboratory experiment no. 63; (**b**) rate of water temperature versus time of ${n}_{seed}$ for the laboratory experiment no. 86; (**c**) rate of water temperature versus time of $E$ for the laboratory experiment no. 63; (**d**) rate of water temperature versus time of $E$ for the laboratory experiment no. 86; (**e**) rate of water temperature versus time of ${n}_{max}$ for the laboratory experiment no. 63; (**f**) rate of water temperature versus time of ${n}_{max}$ for the laboratory experiment no. 86.

In practice, the number of ice crystals ${n}_{seed}$, the diameter $d$ and the aspect ratio of frazil ice $S$ affect each other. Under certain conditions, the increase of the number of ice crystals will limit the growth of ice crystals. Moreover, the roughness of ice crystals will further affect the diameter and the aspect ratio. In turn, it limits the number of ice crystals and other variables until equilibrium is reached. The number of ice crystals is a key parameter to affect the growth of ice crystals in the model. The initial number of ice crystal and the number of nuclei produced are the basic factors affecting the number of ice crystals, which directly determine the starting height. Based on the sensitivity analysis above, the influence of three parameters on the rate of water temperature was analyzed, and the results are shown in Fig. 8.

Figure 8 shows the rate of water temperature versus time is “peak-tip”. Figure 8a–d shows the larger ${n}_{seed}$ and $E$, the smaller the maximum rate of the water temperature. This is because the number of ice crystals has affected the heat exchange between water and ice, so the more there are, the faster the water temperature will recover. On the contrary, Fig. 8e,f shows the maximum rate of water temperature increases with the maximum number of ice crystals ${n}_{\mathrm{max }}$ increase. This may be because increasing ${n}_{\mathrm{max}}$ shorten the steady-state time of water temperature. The frequency and number of collisions between ice and water cannot increase indefinitely, it is quite logical to limit $n$.

In addition, it can also be seen that the influence of ${ n}_{seed}$ is different in different conditions, such as the rate of no. 63 is very small, while no. 86’s rate has a clear gap. Contrast Fig. 7a–d, when the rate of water temperature is equal to 0, the time reaches the maximum cooling. Then the rate of water temperature will have a steeper trend, indicating that it has a significant impact on the stage of warming. Until the water temperature reaches the residual temperature, the rate of water temperature approaches the maximum.

## Conclusion and the future work

In this paper, through defining the processes of secondary nucleation and flocculation by introducing critical impact velocity and the probability of flocculation, the general formula for the number of frazil ice crystals is established and applied the model for frazil ice formation and evolution. The example analysis and sensitivity analysis are carried out for the model. Based on the above, the following conclusions can be drawn:

1. The process of secondary nucleation and flocculation are important for frazil ice formation and evolution. The establishment of general formula for the number of ice crystals can make the model comprehensive and accurate by introducing new parameters.
2. The simulation results agreed favorably with observational data and actual project, and correspond with the theoretical supercooling process. It confirmed that the simulation can reflect the entire physical process of frazil ice formation and evolution.
3. Through the Sobol analysis of the three parameters (${n}_{seed},E, {n}_{\mathrm{max}}$), it can found that the number of nuclei produced $E$ is the most sensitive and has the greatest influence on the calculation of the number of ice crystals and the maximum supercooling.
4. In the sensitivity analysis of the three parameters, it shows that the initial number of ice crystal ${n}_{seed}$ and the number of nuclei $E$ produced directly determine the starting height of the number of ice crystal, which affects the maximum cooling and the period of supercooling. Further nucleation and collision may be restricted during the later stages of supercooling process.

Finally, in the future work, we will strengthen the relevant experiments of physical model and enrich the collection of measured data to obtain the internal relationship among many parameters, So that the parameters can be better calibrated to properly evaluate model.

## Data Availability

The datasets used and/or analyzed during the current study are available from the corresponding author upon reasonable request.

## Abbreviations

DMT: Derjaguin Muller Toporov
JKR: Johnson–Kendall–Roberts
MAE: Mean absolute error
RMSE: Root mean square error
